# The dual role of glycogen synthase kinase-3 beta (GSK3β) in neurodegenerative pathologies: interplay between autophagy and disease progression

**DOI:** 10.3389/fphar.2025.1693805

**Published:** 2025-10-20

**Authors:** Hassan H. Alhassan, Komal Janiyani, Malvi Surti, Mohd Adnan, Mitesh Patel

**Affiliations:** ^1^ Department of Clinical Laboratory Sciences, College of Applied Medical Sciences, Jouf University, Sakaka, Saudi Arabia; ^2^ King Salman Center for Disability Research, Riyadh, Saudi Arabia; ^3^ Research and Development Cell (RDC), Parul University, Vadodara, Gujarat, India; ^4^ Department of Biotechnology, Parul Institute of Applied Sciences, Parul University, Vadodara, Gujarat, India; ^5^ Department of Biology, College of Science, University of Ha’il, Ha’il, Saudi Arabia; ^6^ Department of Computer Science and Bioscience, Faculty of Engineering and Technology, Marwadi University, Rajkot, Gujarat, India

**Keywords:** autophagy, cellular homeostasis, glycogen synthase kinase-3 beta, neurodegenerative diseases, protein aggregation

## Abstract

Glycogen Synthase Kinase-3 Beta (GSK3β), a multifunctional serine/threonine kinase, plays a central role in cellular signaling pathways and autophagy regulation, processes critical to neurodegenerative diseases such as Alzheimer’s disease, Parkinson’s disease, Huntington’s disease and Amyotrophic Lateral Sclerosis (ALS). Dysregulation of autophagy leads to the toxic accumulation of misfolded proteins and damaged organelles, contributing to neuronal loss in these disorders. This review explores the mechanistic interplay between GSK3β and autophagy, highlighting its modulation through key pathways, including mTOR, AMPK and Bcl-2 and its direct impact on autophagy-related proteins such as Beclin-1 and LC3. This review systematically discusses the disease-specific roles of GSK3β in autophagy dysregulation and protein aggregation, providing evidence from recent studies on neurodegenerative models. Additionally, therapeutic approaches targeting GSK3β are evaluated, including preclinical and clinical trials of GSK3β inhibitors and combination therapies with autophagy modulators, emphasizing their potential for improving neuroprotection and cellular homeostasis. Despite its promise, challenges such as off-target effects and pathway complexity remain significant. This review highlights the importance of GSK3β as both a therapeutic target and a biomarker, offering avenues for future research into selective GSK3β modulators that enhance autophagy and mitigate ND progression.

## 1 Introduction

Disorders characterized by the progressive degeneration of nervous system structure and function are collectively known as neurodegenerative diseases (NDs). Among the most prevalent NDs are Alzheimer’s disease (AD), Parkinson’s disease (PD), Huntington’s disease (HD), and amyotrophic lateral sclerosis (ALS). These conditions share common pathological features, including the build-up of abnormal proteins, cellular stress responses, and neuronal death. These shared characteristics are instrumental in the progression of these diseases and the manifestation of their symptoms (Choonara et al., 2009; Arabit et al., 2018; Lamptey et al., 2022; Pircs et al., 2022; Gadhave et al., 2024).

Affecting millions worldwide, AD is the most common type of dementia. The disease is marked by accumulations of amyloid-beta (Aβ) plaques and neurofibrillary tangles of hyperphosphorylated tau protein, resulting in synaptic dysfunction and neuronal loss (Kim et al., 2019; Zetterberg and Blennow, 2021). The onset of AD is often insidious, with cognitive decline and memory impairment being the hallmark symptoms. The disease progresses through stages, ultimately resulting in severe cognitive deficits and loss of independence (Gao Y. et al., 2020). The interplay between Aβ accumulation, neuroinflammation and oxidative stress is critical in the pathogenesis of AD, highlighting the importance of cellular stress responses in neuronal death (Brzecka et al., 2018).

Parkinson’s disease, a common neurodegenerative condition, primarily impacts movement. The defining characteristic of PD is the decline of dopamine-producing neurons within the substantia nigra, which manifests as motor impairments like tremors, stiffness, and slowness of movement (bradykinesia). A key pathological feature of PD is the presence of Lewy bodies, abnormal intracellular clusters of alpha-synuclein protein (Aarsland et al., 2021). Similar to AD, PD is associated with oxidative stress and mitochondrial dysfunction, contributing to neuronal cell death and the progression of motor and non-motor symptoms (Aarsland et al., 2021).

Huntington’s disease is an inherited neurodegenerative disorder stemming from an amplified CAG repeat within the HTT gene. This genetic anomaly leads to the creation of a harmful variant of the huntingtin protein, causing progressive motor difficulties, cognitive deterioration, and psychiatric disturbances (Katzeff et al., 2022). The pathophysiology of HD involves neuronal loss in specific brain regions, particularly the striatum and cortex, with protein aggregation being a central feature of the disease. The toxic effects of mutant huntingtin are intensified by cellular stress responses, including mitochondrial dysfunction and excitotoxicity, which further contribute to neuronal death (Katzeff et al., 2022).

Amyotrophic Lateral Sclerosis involves the decline of both upper and lower motor neurons, progressively weakening muscles and causing them to waste away (atrophy). While the precise causes of ALS remain largely unclear, both genetic predispositions and environmental factors are thought to contribute (Abu-Rumeileh et al., 2020). The disease’s pathology includes the buildup of improperly folded proteins, such as TDP-43, and neuroinflammation, both of which contribute to neuronal demise (Ryberg et al., 2010). The interplay between protein aggregation and cellular stress responses is crucial in ALS, as these factors lead to the disruption of cellular homeostasis and ultimately result in motor neuron degeneration (Ryberg et al., 2010; Cui et al., 2022).

Overall, protein clumping, cellular stress and neuronal death are critically important in the development and progression of neurodegenerative diseases such as Alzheimer’s disease, Parkinson’s disease, Huntington’s disease and ALS. A deeper understanding of these underlying processes is crucial for designing effective treatments to decrease the impact of these neurodegenerative diseases.

## 2 GSK3β - A multifunctional kinase in cellular functions and signaling

**FIGURE 1:**
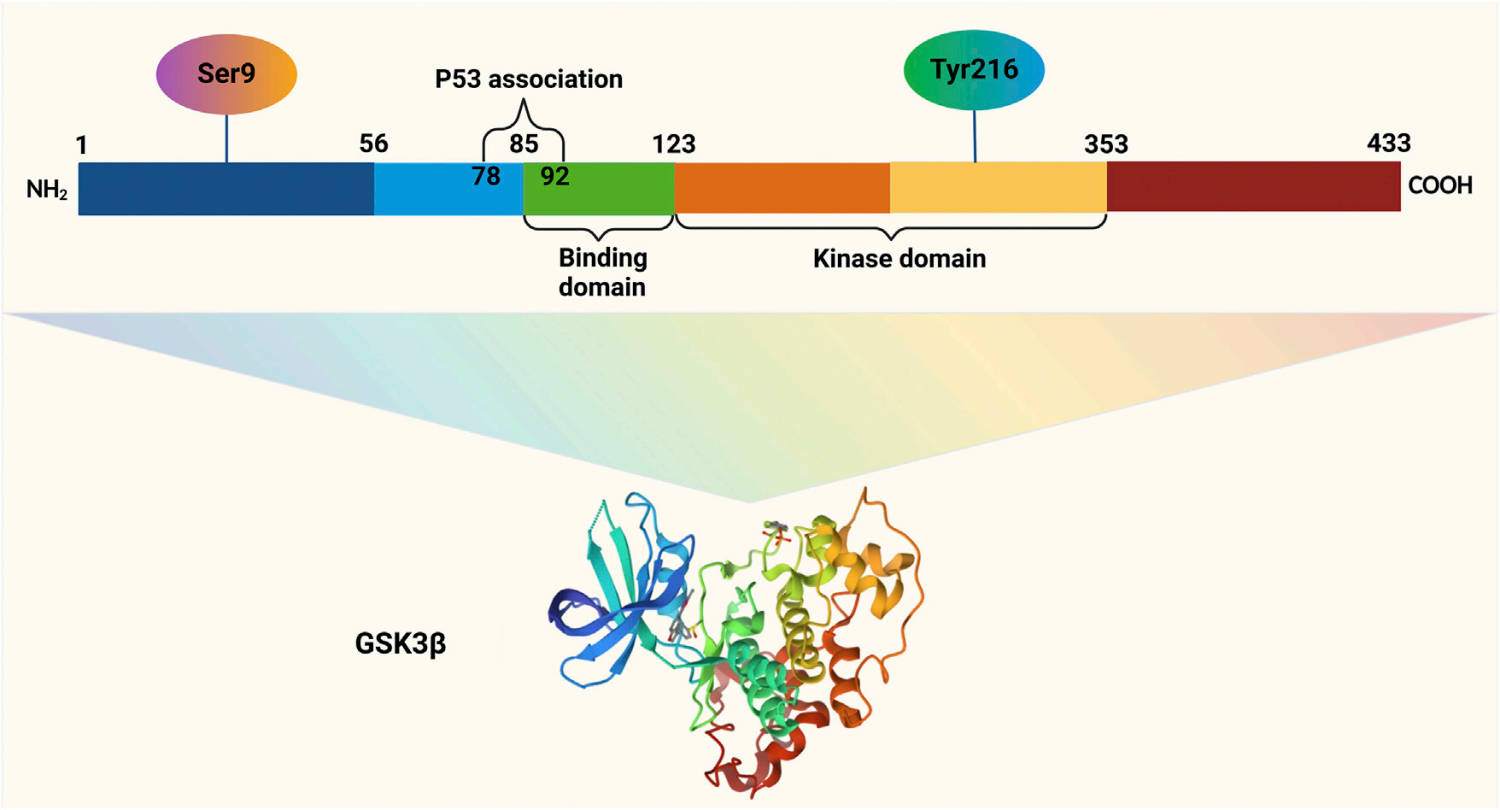
GSK3β, a 47-kDa protein comprising 433 amino acids in humans, is organized into three key regions: the N-terminal domain, the kinase domain, and the C-terminal domain. Activation of GSK3β occurs through phosphorylation at Tyrosine 216 within its N-terminal region, whereas phosphorylation at Serine 9 in the same region leads to its inactivation. The binding domain (BD) of GSK3β facilitates interactions with specific substrates and protein complexes, ensuring its functional versatility.

GSK3β, a serine/threonine kinase, is a key player in numerous cellular processes, encompassing glycogen processing, inflammation and programmed cell death (apoptosis). Initially identified for its role in glycogen synthesis regulation, GSK3β is now recognized as a key player in numerous signaling pathways that influence cell growth, survival and differentiation (Domoto et al., 2020). Its activity is tightly regulated through phosphorylation and dephosphorylation mechanisms, which determine its functional state and influence downstream signaling events (Beurel et al., 2015) (Figure 1). Regarding glycogen metabolism, GSK3β acts as an inhibitor of glycogen synthase, the enzyme that drives glycogen production. This inhibitory action is essential for maintaining balanced glucose levels, especially in reaction to insulin signaling (Domoto et al., 2020). Additionally, GSK3β participates in regulating inflammatory reactions, where its activity can influence the production of pro-inflammatory signaling molecules (cytokines) (Yousef et al., 2022). In the context of apoptosis, GSK3β has been linked to controlling cell death mechanisms, frequently encouraging cell self-destruction in response to cellular stress (Galati et al., 2023).

The regulatory role of GSK3β extends to several critical signaling pathways, notably the Wnt, PI3K/AKT, and mammalian target of rapamycin (mTOR) pathways. Within the Wnt signaling cascade, GSK3β functions as a suppressor. It adds phosphate groups (phosphorylates) to β-catenin, marking it for breakdown and thus preventing the transcription of genes targeted by Wnt signaling. Upon binding of Wnt signaling molecules (ligands) to their corresponding receptors, GSK3β activity is suppressed, enabling β-catenin to build up and move into the cell nucleus, where it triggers gene expression (Chen et al., 2019). This regulation is particularly important in developmental processes and has also implications in cancer biology, where aberrant Wnt signaling is often observed (Abreu de Oliveira et al., 2022).

The PI3K/AKT pathway, vital for cell survival and proliferation, also interacts with GSK3β. AKT inactivates GSK3β by phosphorylating it at serine 9. This inactivation promotes cell survival by inhibiting apoptosis and enhancing cellular growth and metabolism (Kitagishi et al., 2012). Conversely, GSK3β can negatively regulate the PI3K/AKT pathway, creating a feedback loop that is essential for maintaining cellular homeostasis (Galati et al., 2023). GSK3β also has a strong connection to the mTOR pathway, a key control system for cell growth and metabolism. mTOR can inhibit GSK3β activity, while GSK3β can also inhibit mTOR signaling under certain conditions, highlighting a complex interplay between these pathways (Zhang et al., 2015). The regulation of GSK3β within these pathways highlights its significance in cellular responses to different stimuli such as, growth factors and nutrient availability. It is a multifunctional serine/threonine kinase that maintains a number of cellular functions through its involvement in critical signaling pathways, including glycogen metabolism, inflammation and apoptosis. Its regulatory roles in the Wnt, PI3K/AKT and mTOR pathways illustrate its importance in maintaining cellular homeostasis and its capability as a therapeutic target in various diseases, including neurodegenerative disorders and cancer.

## 3 Autophagy: essential for homeostasis and its role in neurodegenerative diseases

A highly conserved cellular process called autophagy is essential for maintaining cellular balance (homeostasis). It achieves this by degrading damaged organelles, misfolded proteins, and other cellular waste. This process involves the creation of double-membrane vesicles, termed autophagosomes, which enclose the targeted cellular components before fusing with lysosomes for breakdown (Rubinsztein et al., 2012; Rahman and Rhim, 2017). The resulting breakdown products, including amino acids and fatty acids, are recycled back into the cell’s interior (cytoplasm) to fuel vital biosynthetic processes, thereby supporting cell survival and function during stressful times (Lynch-Day et al., 2012; Aman et al., 2021). Beyond its role in cellular cleanup, autophagy is essential for a range of physiological processes, including cellular differentiation, development and the cellular response to nutrient shortage (Aman et al., 2021). Impaired autophagy, a key factor in several diseases, plays a significant role in neurodegenerative disorders such as AD, PD, HD, and ALS. In these conditions, the resulting accumulation of toxic protein aggregates and damaged organelles contributes to neuronal dysfunction and cell death (Rahman and Rhim, 2017; Guo et al., 2018).

A key characteristic of neurodegenerative diseases is the failure of autophagy, leading to the build-up of misfolded proteins such as amyloid-beta (in AD) and alpha-synuclein (in PD). These proteins are known to form harmful aggregates that interfere with normal cellular processes (Palmer et al., 2024). For example, in AD, extensive autophagic pathology is observed, characterized by the accumulation of autophagic vacuoles in affected neurons, which arises from impaired clearance mechanisms (Tizon et al., 2010). In PD, abnormal lysosomal activity and autophagy dysfunction cause misfolded alpha-synuclein to aggregate, leading to neuronal death (Bayati and McPherson, 2024).

Research indicates that boosting autophagy can protect neurons, suggesting that therapies targeting autophagic activity could be beneficial for neurodegenerative diseases (Djajadikerta et al., 2020; Talebi Taheri et al., 2024). For example, small molecules that stimulate autophagy have been shown to enhance the removal of damaged cellular components and improve neuronal survival in Huntington’s disease models (Tsvetkov et al., 2010). Conversely, genetic alterations affecting proteins involved in autophagy, like ATG5, have been associated with neurodegenerative characteristics, highlighting autophagy’s crucial role in neuronal health (Kim et al., 2016). Therefore, autophagy is a vital cellular degradation process that maintains cellular balance by eliminating damaged organelles and misfolded proteins. Autophagy dysfunction significantly contributes to the development of neurodegenerative diseases, where impaired autophagy leads to the build-up of toxic protein clumps and ultimately neuronal death. Investigating the mechanisms behind autophagy dysregulation may offer valuable knowledge for developing potential treatments for these chronic conditions.

## 4 Linking GSK3β and autophagy in neurodegenerative diseases

GSK3β’s potential to affect neurodegeneration through autophagy regulation is attracting significant attention in neurobiology. Its dysregulation is implicated in the development of various neurodegenerative diseases, such as AD, PD, HD, and ALS (Reddy, 2013; Guo et al., 2018). The relationship between GSK3β and autophagy is highly relevant, given that defective autophagic processes can result in the accumulation of toxic protein aggregates and damaged organelles, hallmarks of neurodegenerative diseases (Ghavami et al., 2014).

This review is intended to analyze the function of GSK3β in autophagy regulation and its potential impact on neurodegenerative diseases. A better understanding of how GSK3β affects autophagy could reveal insights into the mechanisms driving neurodegeneration and point to potential therapeutic targets. Research has demonstrated that GSK3β can impact autophagy by regulating crucial autophagy-related proteins and pathways, including mTOR and Wnt signaling (Golpich et al., 2017). Furthermore, involvement of GSK3β in oxidative stress responses may also affect autophagy, as oxidative stress is known to impair autophagic function and contribute to neuronal cell death (Reddy, 2013; Guo et al., 2018).

Importantly, GSK-3β’s function is tightly regulated by its phosphorylation state; it remains active when phosphorylated at tyrosine 216 (Tyr216) and is inhibited when phosphorylated at serine 9 (Ser9) (Gao J. et al., 2020). However, it is important to note that autophagy induction is only one mechanism associated with neuronal survival following ischemic brain injury. Neuronal death can also occur through necrosis or necroptosis, characterized by cytoplasmic swelling and membrane rupture (Vandenabeele et al., 2010), and apoptosis, which involves cell shrinkage, nuclear condensation, mitochondrial damage, membrane blebbing, and DNA fragmentation (Nikoletopoulou et al., 2013). Although these processes are mechanistically distinct, they share molecular mediators and regulatory hubs, such as Bcl-2, AMP-activated protein kinase (AMPK), and p62, which integrate signaling pathways controlling autophagy, apoptosis, and necroptosis (Nikoletopoulou et al., 2013; Levine et al., 2015). These integrative hubs coordinate protein complex formation, membrane trafficking, and metabolic sensing, thereby linking multiple cell death and survival pathways in neurons (Pluta, 2023).

In Alzheimer’s disease models, aberrant activation of GSK-3β has been associated with tau hyperphosphorylation, disrupting microtubule stability and thereby accelerating neurodegeneration (Li et al., 2014; Jin et al., 2015). This highlights that GSK-3β not only regulates autophagy but also directly contributes to tau pathology. Interestingly, GSK-3β exhibits context-dependent roles in autophagy. Under ischemic stress, its activation can promote autophagic clearance of damaged proteins and organelles, providing neuroprotection (Gao J. et al., 2020). In contrast, under energy-rich conditions, GSK-3β activates the mTOR pathway, which suppresses autophagy through the downregulation of essential autophagy-related proteins (Liu et al., 2015; Jiang et al., 2018; Pan and Valapala, 2022). The interplay with AMPK signaling further refines this regulation: activation of AMPK can inhibit GSK-3β, thereby enhancing autophagy and supporting neuronal health, particularly in Parkinson’s disease models where AMPK-mediated suppression of GSK-3β reduces neuroinflammation (Li et al., 2014; Sun et al., 2016; Duan et al., 2019).

Moreover, GSK-3β influences autophagy at the transcriptional level through its regulation of transcription factor EB (TFEB), a master regulator of lysosomal biogenesis and autophagy-related gene expression. Misregulation of this pathway disrupts autophagic flux and lysosomal function, leading to accumulation of toxic cellular debris and further amplifying neurodegenerative pathology (Pan and Valapala, 2022).

In neurodegenerative diseases, a detrimental feedback loop may arise from the interplay between GSK3β and autophagy. Impaired autophagy leads to a build-up of misfolded proteins, which in turn further activate GSK3β, thus worsening neurodegeneration (Ghavami et al., 2014). Collectively, the evidence highlights GSK-3β as a multifaceted regulator of autophagy through phosphorylation-dependent activity, cross-talk with AMPK and mTOR signaling, and modulation of transcriptional programs such as TFEB. Its dual role in promoting or suppressing autophagy, depending on cellular context, highlights its therapeutic potential as a target to restore autophagic balance and slow neurodegenerative progression (Gao J. et al., 2020; Pan and Valapala, 2022). By clarifying this complex relationship, this review aims to demonstrate the therapeutic potential of targeting GSK3β to improve autophagy and slow the progression of these diseases.

## 5 Activation and inhibition of GSK3β

GSK3β is critically regulated through phosphorylation and dephosphorylation processes. The regulation of GSK3β is complex, involving multiple signaling pathways that determine its activity and consequently, its role in several cellular functions with metabolism, cell survival and autophagy.

### 5.1 Regulation through phosphorylation and dephosphorylation

**FIGURE 2:**
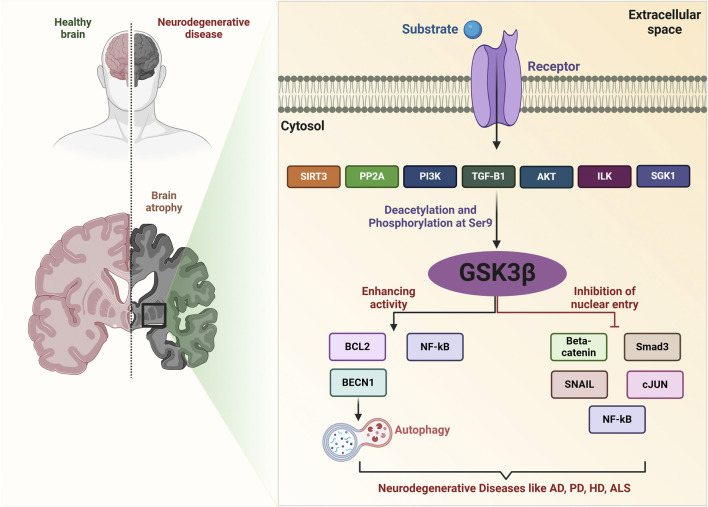
GSK-3β signaling network in neurodegenerative diseases. SIRT3 deacetylates GSK-3β in mitochondria, destabilizing substrates such as SMAD3 and c-Jun, which reduces their nuclear import. The PI3K/AKT signaling pathway phosphorylates GSK-3β at Ser9, leading to its inactivation. SGK1 can also inactivate GSK-3β through phosphorylation. TGF-β1 increases the expression of Ser9-phosphorylated, inactive GSK-3β. PP2A induces GSK-3β expression, regulating various processes. Activated ILK increases Ser9 phosphorylation of GSK-3β, associated with various pathological processes. GSK-3β also mediates phosphorylation of substrates like SNAIL, BCL2, β-Catenin, SMAD3, c-Jun, and NF-κB, inhibiting their function. Key molecules include ROS (reactive oxygen species), SIRT3, PI3K, Akt, SGK1, TGF-β1, PP2A, ILK, and other related proteins.

The activity of GSK3β is primarily modulated through phosphorylation at specific residues. Phosphorylation at serine 9 (Ser9) by protein kinase B (Akt) results in the inactivation of GSK3β, whereas phosphorylation at tyrosine 216 (Tyr216) serves to augment its activity (Bardai and D'Mello, 2011; Ghanaatfar et al., 2023). When GSK3β is phosphorylated at Ser9, it undergoes a conformational change that inhibits its kinase activity, preventing it from phosphorylating its substrates, such as glycogen synthase and various transcription factors (Kong et al., 2019). Conversely, the phosphorylation at Tyr216 activates GSK3β, promoting its role in signaling pathways that can lead to neurodegeneration (Yang et al., 2012). Dephosphorylation of GSK3β can occur through the action of phosphatases, which can reverse the inhibitory phosphorylation at Ser9, thereby reactivating GSK3β. Cells rely on a dynamic equilibrium between phosphorylation and dephosphorylation to maintain homeostasis and respond to a range of stimuli, including growth factors and stress signals (Wei et al., 2023) (Figure 2).

### 5.2 Activation and inhibition through signaling pathways

**FIGURE 3:**
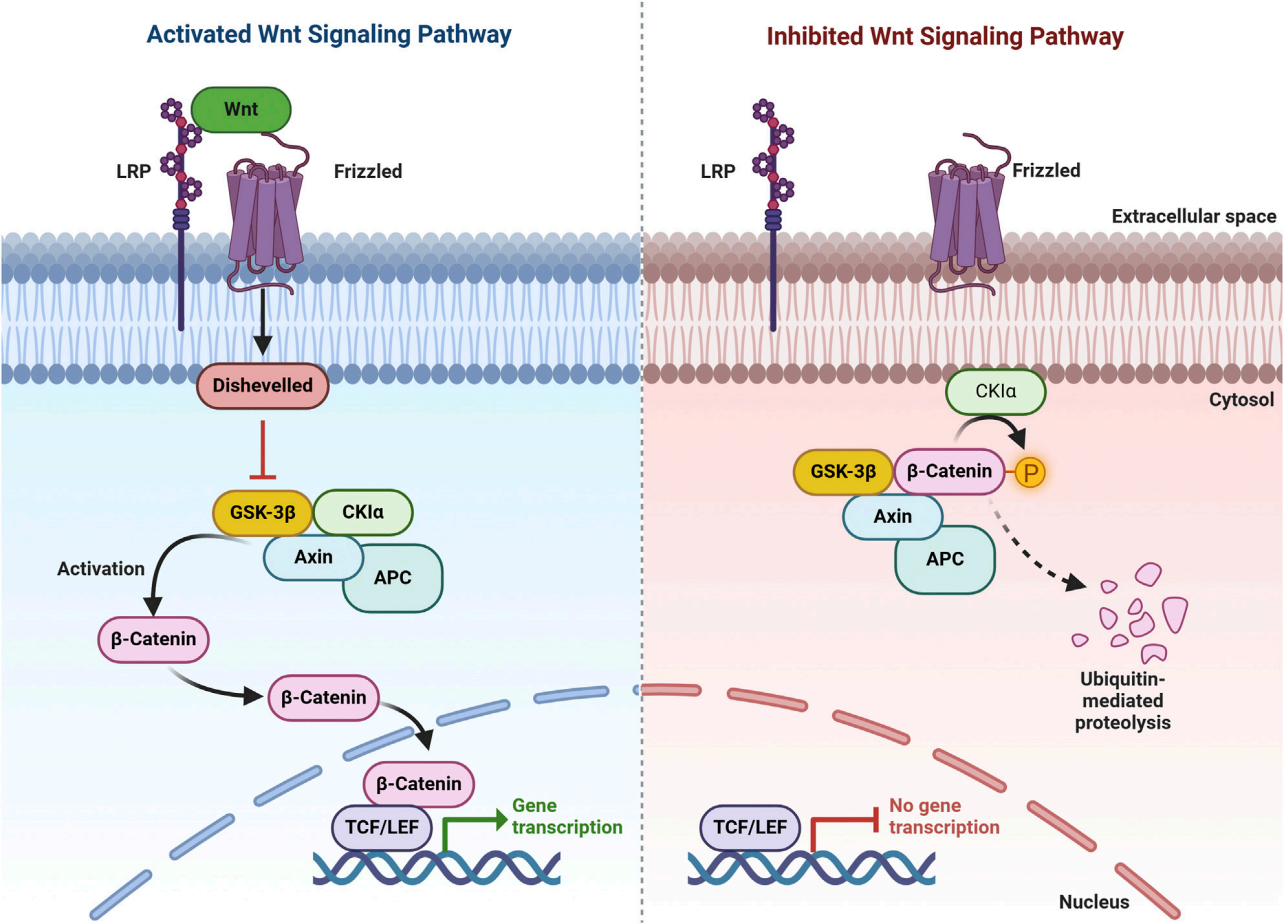
Wnt/β-Catenin Pathway. In the presence of Wnt signaling (activating pathway), Wnt binds to its receptor, promoting the recruitment of AXIN to phosphorylated lipoprotein receptor-related protein (LRP). This disrupts the destruction complex, stabilizes β-catenin, and enables its translocation to the nucleus, where it binds to TCF to regulate target gene expression. In the absence of Wnt signaling (inhibiting pathway), β-catenin is targeted for degradation by the destruction complex, which includes AXIN, APC, serine/threonine kinase GSK-3, CK1, and E3 ubiquitin ligase β-TrCP.

Several important signaling pathways, including PI3K/AKT, Wnt, and AMPK signaling, regulate GSK3β. The PI3K/AKT pathway is particularly well-understood in this regard. When growth factors activate PI3K, it phosphorylates and activates Akt, which then phosphorylates GSK3β at Ser9, causing its inactivation (Bardai and D'Mello, 2011; Ghanaatfar et al., 2023). This inhibition of GSK3β is crucial for promoting cell survival and preventing apoptosis, particularly in neuronal cells. In conditions of cellular stress or neurodegeneration, the dysregulation of this pathway can lead to increased GSK3β activity, contributing to neuronal death (Liu et al., 2021). A key component of the Wnt signaling pathway is GSK3β. In the absence of Wnt ligands, GSK3β phosphorylates β-catenin, targeting it for degradation and preventing Wnt target gene transcription. Conversely, Wnt ligand binding to receptors inhibits GSK3β, enabling β-catenin accumulation and nuclear translocation to activate gene expression (Badimon et al., 2019; Pan and Valapala, 2022). This regulatory mechanism is vital for cellular processes like proliferation and differentiation, and its disruption can contribute to conditions like cancer and neurodegeneration (Figure 3). Another important GSK3β regulator is AMPK. Under conditions of energy stress, AMPK is activated and can inhibit mTORC1, which in turn affects GSK3β activity. AMPK can also directly phosphorylate GSK3β, leading to its inhibition and promoting autophagy (Ren et al., 2016; Kong et al., 2019). This pathway highlights the action of GSK3β in cellular energy metabolism and its potential impact on autophagic processes. In addition to the pathways already mentioned, GSK3β is also regulated by other signaling networks, such as those involved in cytokine signaling and stress responses. For example, inflammatory cytokines can activate GSK3β, leading to increased neuronal injury in neurodegenerative diseases (Zhou et al., 2018). Additionally, various pharmacological agents, such as lithium, have been shown to inhibit GSK3β activity, providing a potential therapeutic avenue for conditions characterized by GSK3β dysregulation (Dedert et al., 2023; Ghanaatfar et al., 2023).

## 6 Autophagy regulation pathways and GSK3β

GSK3β plays a significant role in the regulation of autophagy, a cellular degradation process essential for sustaining cellular homeostasis. GSK3β is known to influence autophagy through multiple mechanisms. A key pathway involved is the activation of AMPK. Inhibition of GSK3β has been shown to activate AMPK, which subsequently promotes autophagy by inhibiting mTOR, a key negative regulator of autophagy (Wang et al., 2019). For example, in goat muscle satellite cells, GSK3β inhibition led to increased autophagic activity through the AMPK pathway, highlighting the importance of GSK3β in modulating autophagy in muscle cells (Wang et al., 2019). Similarly, in models of liver failure, GSK3β inhibition was associated with enhanced autophagy, indicating its protective role in cellular stress conditions (Wang et al., 2019). Moreover, GSK3β is involved in the regulation of ULK1, a critical initiator of autophagy. GSK3β can phosphorylate ULK1, thereby influencing its activity and the subsequent formation of autophagosomes (Ryu et al., 2021). This regulation is particularly relevant under stress conditions, where autophagy is activated to promote cell survival.

The mTOR pathway is another critical regulator of autophagy and GSK3β plays a dual role in this context. GSK3β can inhibit mTOR activity, thereby promoting autophagy (Kong et al., 2019). Conversely, mTOR can also phosphorylate GSK3β, leading to its inactivation. This reciprocal regulation creates a complex feedback loop where mTOR activity influences GSK3β, which in turn affects autophagy (Kong et al., 2019). For example, rapamycin, an mTOR inhibitor, has been shown to alter the Wnt/GSK3β/β-catenin signaling pathway, further emphasizing the interconnectedness of these pathways in regulating autophagy (Chen et al., 2019). GSK3β also plays a key regulatory role in the Wnt signaling pathway. When Wnt signaling is absent, GSK3β promotes the phosphorylation and breakdown of β-catenin, a crucial transcription factor for cell growth and specialization. Conversely, when Wnt signals bind to their receptors, GSK3β is inhibited, allowing β-catenin to build up and activate target genes (Li et al., 2023). This control is essential for cellular function, and disruptions to this pathway can cause autophagy problems and contribute to various diseases, including cancer and neurodegenerative disorders (Shi, 2022; Li et al., 2023).

**FIGURE 4:**
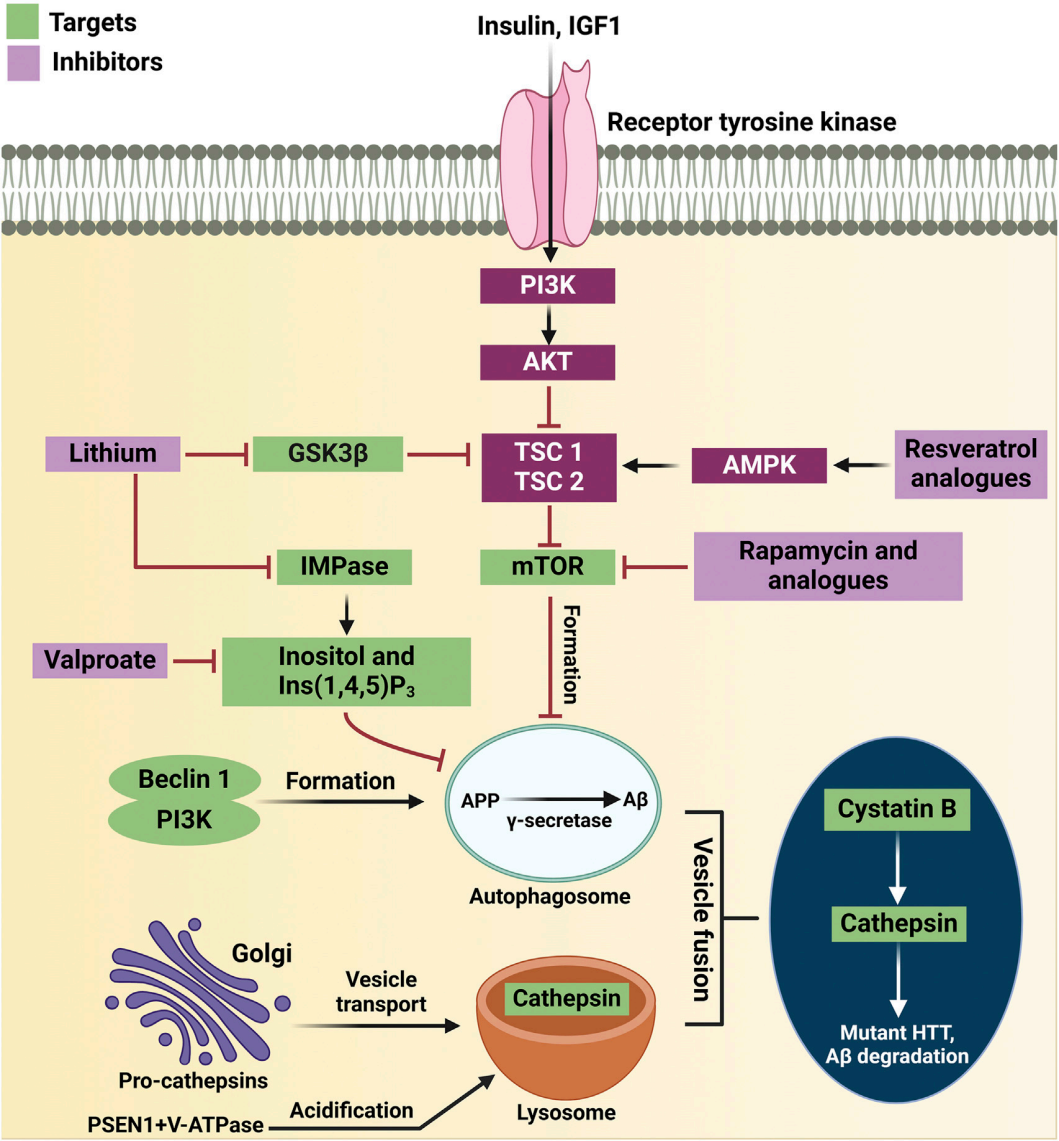
Therapeutic targets in the autophagy pathway for neurodegenerative diseases. Resveratrol analogs activate AMPK, promoting autophagy by shifting the balance from AKT inhibition. Lithium inhibits GSK3β, preventing tau phosphorylation and enhancing CREB signaling, which boosts autophagy. Rapamycin and analogs inhibit mTOR, allowing autophagosome formation. Valproate activates an mTOR-independent autophagy pathway by reducing inositol levels. The Beclin 1-PI3K complex supports autophagosome formation, and its inhibition worsens disease conditions. Autophagosomes clear toxic proteins, and fusion with lysosomes enables degradation by cathepsins. Cystatin B deletion improves autophagy, while mutant huntingtin and PSEN1 mutations impair lysosomal function, reducing protein degradation. Key proteins include APP, IGF1, TSC1, and V-ATPase.

Overall, GSK3β is a pivotal regulator of autophagy, influencing various signaling pathways such as AMPK, mTOR and Wnt. Its ability to modulate autophagic activity through these pathways underscores its importance in cellular homeostasis and highlights its potential as a therapeutic target in autophagy-related diseases. Understanding the link between GSK3β and autophagy could inform the development of treatments for neurodegenerative diseases and other autophagy-related conditions. GSK3β is a critical regulator of autophagy, influencing various autophagy-related proteins and pathways that are essential for maintaining cellular homeostasis. In neurodegenerative diseases, the GSK3β-protein interaction is crucial, as autophagy impairment contributes to toxic aggregate accumulation and neuronal death (Figure 4).

## 7 Influence of GSK3β on autophagy-related proteins

### 7.1 ULK1 (Unc-51 like autophagy activating kinase 1)

GSK3β phosphorylates ULK1, a key initiator of autophagy, thereby modulating its activity. This phosphorylation can inhibit ULK1’s function, leading to reduced autophagic activity. Inhibition of GSK3β has been shown to enhance ULK1 activity, promoting autophagy under stress conditions (Yang et al., 2012; Wei et al., 2023). In models of neurodegeneration, GSK3β inhibition enhances autophagic flux and promotes cell survival, according to research (Wei et al., 2023).

### 7.2 Beclin1

Beclin1 is another critical protein involved in the formation of autophagosomes. GSK3β can phosphorylate Beclin1, which affects its role in autophagy initiation. Reduced Beclin1 levels have been associated with neurodegenerative diseases, indicating that GSK3β’s regulation of proper autophagic function depends on Beclin1 (Weikel et al., 2016). In Alzheimer’s disease models, the build-up of toxic amyloid-beta peptide aggregates has been associated with dysregulation of both Beclin1 and GSK3β (Weikel et al., 2016).

### 7.3 LC3 (microtubule-associated protein 1A/1B-light chain 3)

Essential for autophagy, LC3 is a well-established autophagosome marker. GSK3β influences the LC3-I to LC3-II conversion, vital for autophagosome formation. Research shows GSK3β inhibition increases LC3-II, indicating enhanced autophagy (Kong et al., 2019; Dedert et al., 2023). This relationship is crucial in neurodegenerative diseases, where impaired autophagy results in toxic protein aggregate accumulation, reflecting disrupted autophagic flux (Dedert et al., 2023).

### 7.4 p62/SQSTM1

As a selective autophagy receptor, p62 is a multifunctional protein, linking ubiquitinated substrates to the autophagic machinery. GSK3β influences the levels of p62 and its dysregulation can lead to p62 accumulation, indicating impaired autophagic flux (Ren et al., 2016). In neurodegenerative diseases, elevated p62 levels are often observed, correlating with autophagic dysfunction and contributing to neuronal cell death (Ren et al., 2016).

Targeting GSK3β offers therapeutic promise for neurodegenerative diseases due to its crucial role in regulating autophagy proteins. GSK3β inhibition boosts autophagy and promotes toxic protein aggregate clearance, potentially mitigating Alzheimer’s disease and Parkinson’s disease progression (Gu et al., 2019; Wang et al., 2019). For example, pharmacological agents that inhibit GSK3β have demonstrated neuroprotective effects in preclinical models, underscoring the potential of this approach in developing novel treatments for neurodegenerative disorders (Gu et al., 2019; Wang et al., 2019). Hence, GSK3β plays a pivotal role in the regulation of autophagy-related proteins, influencing the autophagic process and its implications in neurodegenerative diseases. Understanding how GSK3β interacts with these proteins is essential for developing therapies to enhance autophagy in neurodegeneration.

## 8 Role of GSK3β-mediated autophagy in specific neurodegenerative diseases

### 8.1 GSK3β and autophagy in Alzheimer’s disease

GSK3β is a key regulator of autophagy, especially in Alzheimer’s disease. This enzyme influences cellular metabolism and survival, processes vital in neurodegenerative conditions. Inhibiting GSK3β activates autophagy, promoting the removal of amyloid-beta (Aβ) peptides, a central component of AD pathology (Kerr et al., 2017; Kong et al., 2019). Specifically, GSK3β inhibition can boost autophagic flux, reducing Aβ build-up and improving neuronal health (Kerr et al., 2017). Research indicates that dysfunctional autophagy plays a significant role in AD development. Autophagy degrades misfolded proteins and damaged organelles, including mitochondria, which are frequently impaired in AD (Kerr et al., 2017; Baig et al., 2023). Impaired autophagy can lead to Aβ and tau protein accumulation, worsening neurodegeneration (Li et al., 2010). For example, studies have shown that presenilin-1 mutations, affecting the γ-secretase complex, disrupt autophagic flux and correlate with increased Aβ levels (Lee et al., 2010; Yang et al., 2019). These findings suggest a crucial link between GSK3β, autophagy, and Aβ metabolism in understanding AD.

**FIGURE 5:**
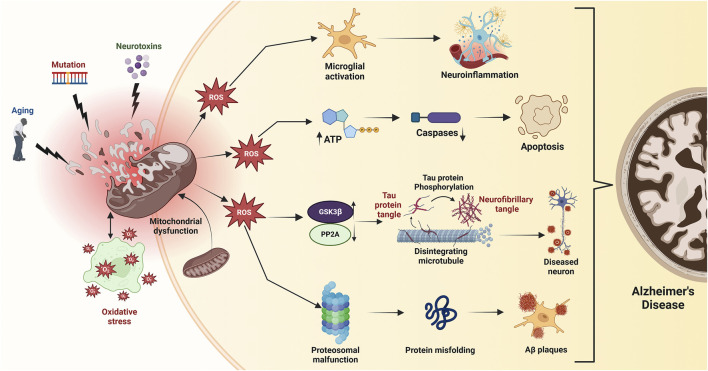
Mitochondrial dysfunction, triggered by age-related factors, mutations, and toxic exposures, leads to bioenergetic deficits, calcium imbalance, and free radical production, resulting in oxidative stress. Excessive ROS disrupts mitochondrial membrane potential (ΔΨm), reduces ATP production, and activates caspases, initiating apoptosis. Elevated ROS also inhibits protein phosphatase 2A (PP2A), activating GSK3β which promotes tau hyperphosphorylation and neurofibrillary tangle accumulation, exacerbating Alzheimer’s disease.

The role of autophagy in AD is nuanced, as both excessive and insufficient activity can worsen the disease. While enhanced autophagy can facilitate the degradation of Aβ, it can also inadvertently activate the amyloidogenic pathway under certain conditions, leading to increased Aβ production (Funderburk et al., 2010; Hung and Livesey, 2018). This duality points out the necessity for a balanced autophagic response, which is modulated by GSK3β activity. Inhibition of GSK3β not only promotes autophagy but also mitigates the adverse effects of dysregulated autophagic processes, thereby providing a potential therapeutic target for AD (Kerr et al., 2017; Kong et al., 2019). GSK3β is therefore a crucial autophagy regulator in AD, influencing neurotoxic protein clearance and neuronal health. This intricate connection between GSK3β, autophagy, and amyloid pathology emphasizes the need to target these pathways in AD therapeutic development (Figure 5).

### 8.2 GSK3β and autophagy in Parkinson’s disease

In PD, GSK3β dysregulation is linked to α-synuclein accumulation, a key feature of the disease. α-synuclein is primarily cleared via autophagy (Li et al., 2018; Parekh et al., 2019). While some studies suggest that GSK3β activation can enhance autophagy and α-synuclein degradation (Li et al., 2018; Parekh et al., 2019), other research indicates that GSK3β inhibition can decrease autophagic flux, leading to toxic protein aggregate accumulation and worsened neurodegeneration (Sun et al., 2016). Notably, GSK3β inhibition can promote autophagy through AMPK pathway activation, crucial for cellular energy balance and autophagy regulation (Sun et al., 2016). This suggests that targeting GSK3β could therapeutically enhance α-synuclein clearance and mitigate PD pathology.

**FIGURE 6:**
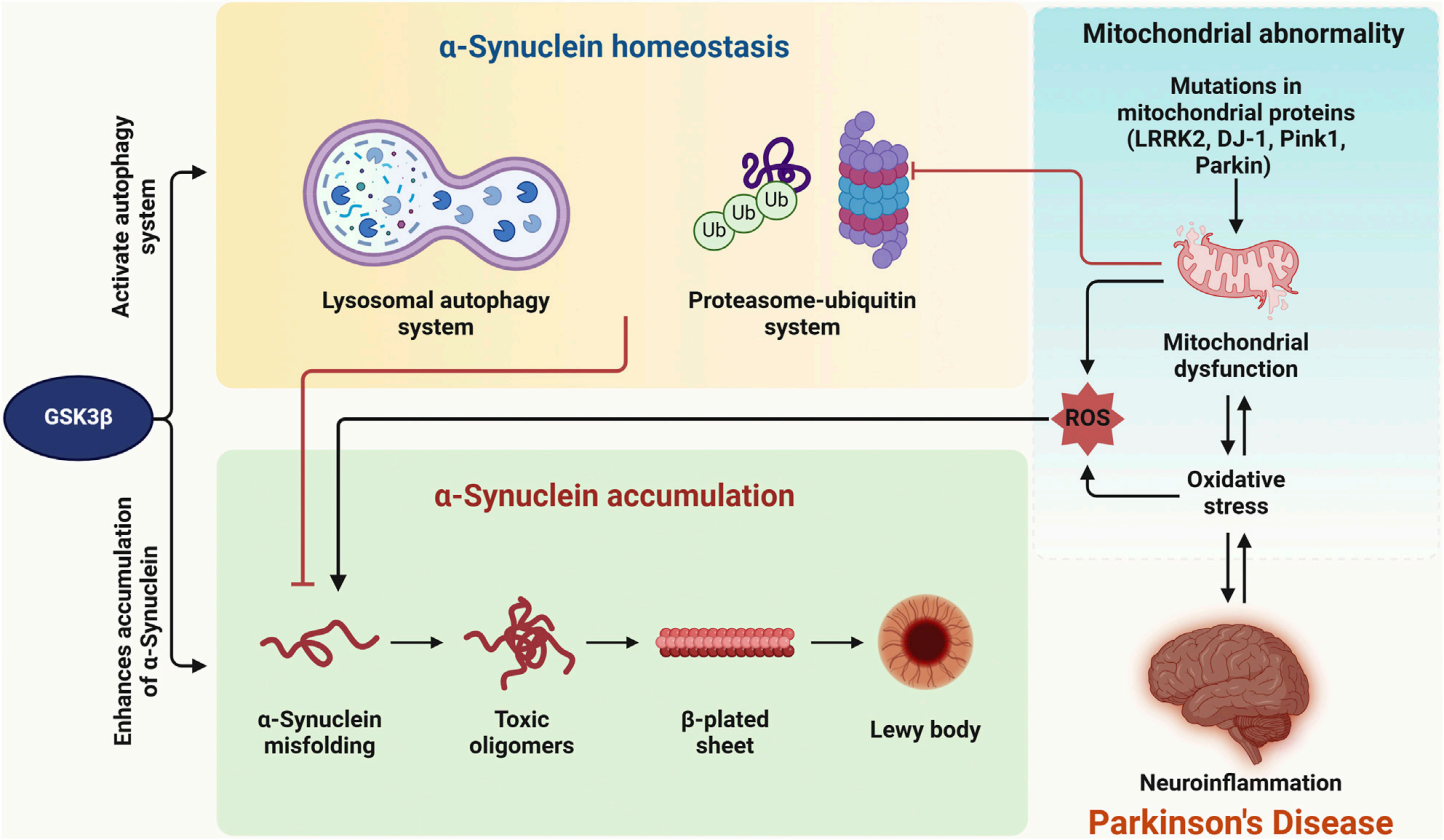
Intracellular α-synuclein homeostasis is maintained *via* the ubiquitin-proteasome and lysosomal autophagy systems, both of which are regulated by GSK3β. Dysregulation of GSK3β directly leads to the accumulation of α-synuclein. Impairment of these degradation systems by oxidative stress (OS), mitochondrial dysfunction, or neuroinflammation could further contribute to α-synuclein accumulation. Additionally, mutations in genes such as LRRK2, DJ-1, Parkin, and Pink1 cause mitochondrial dysfunction and increase cell death. OS and neuroinflammation appear to be interconnected, ultimately resulting in the pathogenesis of Parkinson’s disease.

However, the GSK3β-autophagy relationship is complex and context-dependent. Although GSK3β can promote autophagy, excessive activation can trigger cellular stress and apoptosis, emphasizing the need for balanced autophagy (Lu et al., 2021). In PD, impaired autophagy contributes to misfolded protein accumulation and mitochondrial dysfunction (Wei et al., 2016; Parekh et al., 2019). This inhibition is often associated with lysosomal dysfunction, where GSK3β also plays a regulatory role (Li et al., 2018). Furthermore, the interplay between GSK3β and other pathways, like mTOR, further complicates autophagy regulation in PD. GSK3β can act both upstream and downstream of mTOR, influencing autophagy based on cellular conditions (Lu et al., 2021). This dual role highlights GSK3β′s therapeutic potential, as modulating its activity could restore autophagy and improve neuronal health in PD (Sun et al., 2016). Therefore, GSK3β is a crucial autophagy regulator in PD, influencing the breakdown of α-synuclein and other toxic aggregates. Modulating GSK3β activity offers a promising approach to enhance autophagy and potentially decrease the neurodegenerative effects of PD (Figure 6).

### 8.3 GSK3β and autophagy in Huntington’s disease

GSK3β is a key autophagy regulator, and its role in HD has received considerable research attention. HD is characterized by mutant huntingtin (mHtt) protein accumulation, leading to neurodegeneration, primarily in the striatum and cortex. mHtt aggregation is a central pathological hallmark of HD, and autophagy is crucial for clearing these toxic aggregates (Pircs et al., 2022). Evidence suggests that GSK3β modulates autophagy in HD. Inhibiting GSK3β enhances autophagy, promoting mHtt degradation and reducing its toxicity (Zhang et al., 2015). For example, mTOR inhibition, which activates autophagy, can reduce toxicity from polyglutamine expansions in HD models (Ravikumar et al., 2004), suggesting GSK3β may mediate mTOR signaling, influencing autophagic responses to mHtt (Arabit et al., 2018).

**FIGURE 7:**
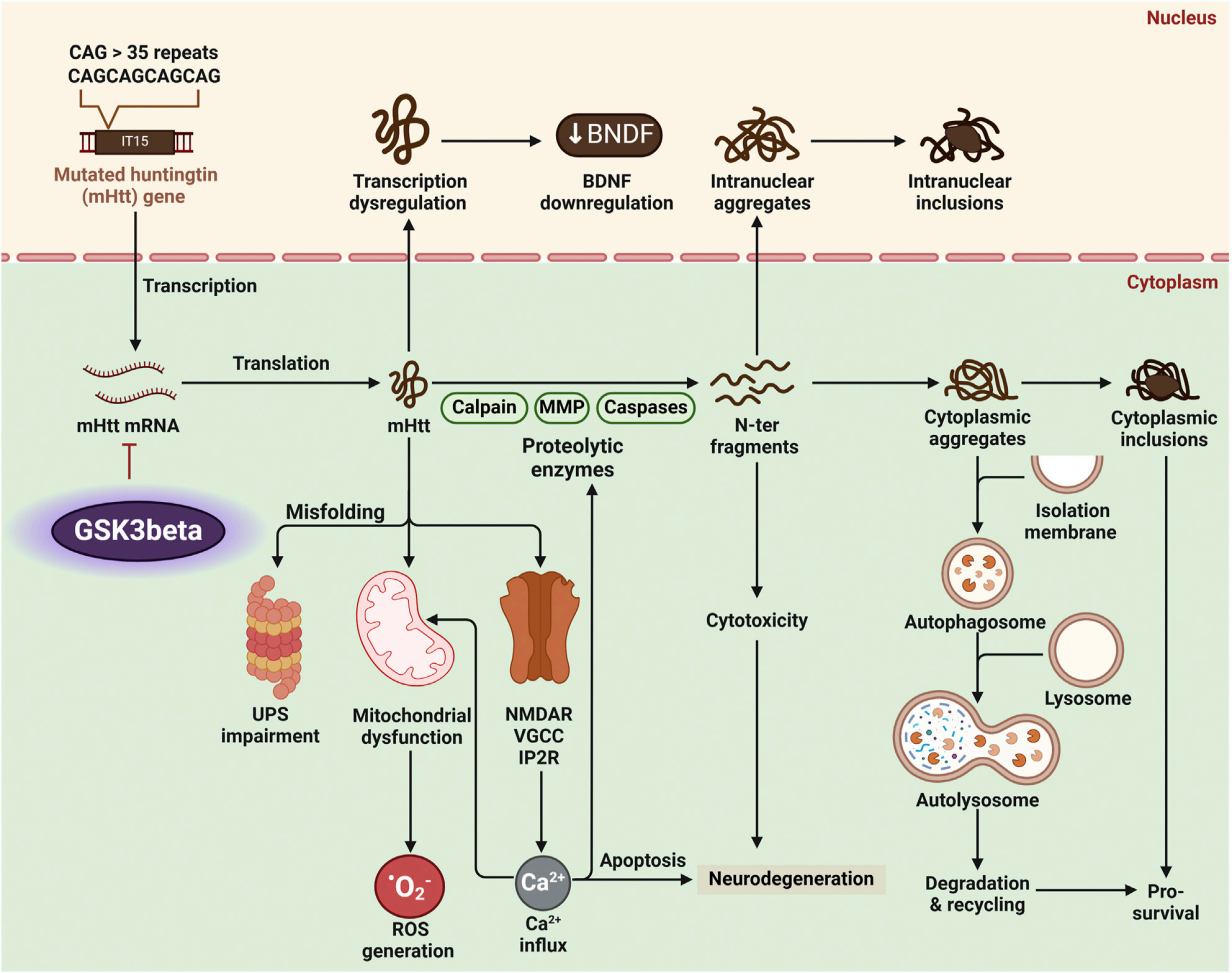
Huntington’s disease (HD) pathology arises from the production of mutant Huntingtin protein (mHtt) with an expanded polyglutamine (polyQ) tract. Proteolytic enzymes like calpains and caspases process mHtt into toxic N-terminal fragments, forming inclusion bodies in neurons. Dysregulated glycogen synthase kinase 3 beta (GSK3β) activity amplifies HD pathology by promoting mitochondrial dysfunction, impairing autophagy, and enhancing mHtt aggregation and toxicity. These processes lead to oxidative stress, neuronal apoptosis, and neurodegeneration.

Furthermore, levels of autophagy-related proteins like Beclin-1 (*BECN1*) decline with age in HD patients, suggesting a link between GSK3β activity and autophagy dysfunction (Pircs et al., 2022). *BECN1* overexpression can slow HD progression, highlighting autophagy’s importance for neuronal health (Pircs et al., 2022). Further supporting GSK3β′s role in autophagy regulation, research suggests it influences autophagosome formation, essential for degrading aggregated proteins (Wold et al., 2016). Moreover, reduced autophagy in HD models correlates with increased neurodegeneration, suggesting enhanced autophagic flux could be therapeutic (Arabit et al., 2018). For example, GSK3β-inhibiting drugs have improved autophagy and reduced mHtt aggregates in HD cell models (Sarkar et al., 2005). This highlights the potential of targeting GSK3β to restore autophagy and protect neurons in HD (Figure 7).

### 8.4 GSK3β and autophagy in amyotrophic lateral sclerosis

GSK3β is also a key regulator of autophagy in ALS, a disease marked by progressive motor neuron degeneration, leading to muscle weakness and atrophy. Recent research highlights GSK3β′s role in modulating autophagy, crucial for neuronal survival and function in ALS (Zhang et al., 2015; Wei et al., 2023). GSK3β influences autophagy through various signaling pathways, including regulation by phosphorylation: Ser9 phosphorylation inhibits its activity, while Tyr216 phosphorylation activates it (Yang et al., 2012). In ALS, GSK3β dysregulation can impair autophagic flux, contributing to misfolded protein accumulation and cellular stress (Chong et al., 2018). This accumulation is particularly damaging in ALS, where the aggregation of proteins like superoxide dismutase 1 (SOD1) is a hallmark (Zhang et al., 2015). Inhibiting GSK3β enhances autophagy, improving toxic protein aggregate clearance in ALS cell models (Kong et al., 2019). For instance, pharmacological GSK3β inhibition can promote autophagy by activating the AMPK pathway, crucial for energy balance and stress responses (Kong et al., 2019). This suggests targeting GSK3β could be a viable therapeutic approach to enhance autophagic clearance and protect motor neurons in ALS.

**FIGURE 8:**
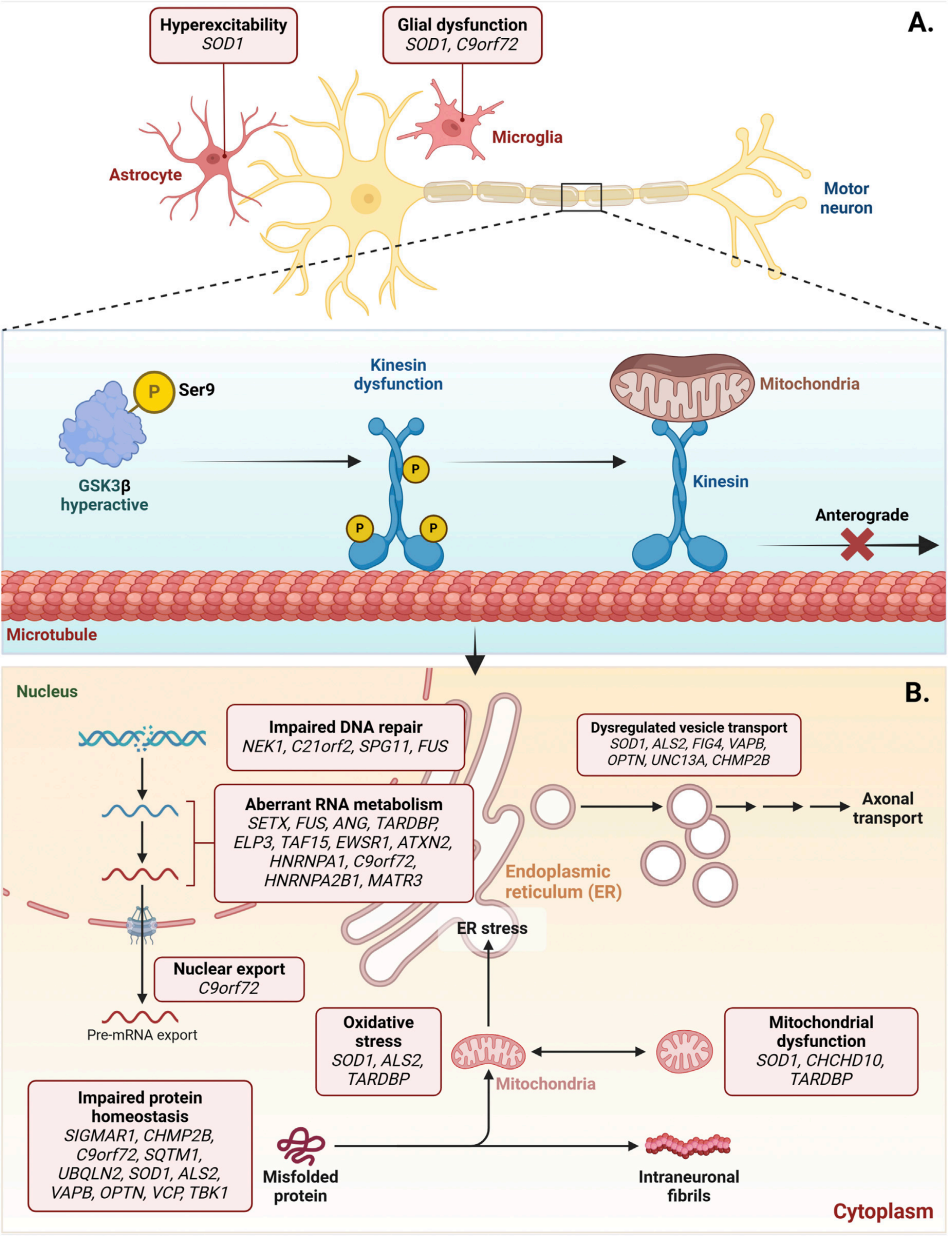
Dysregulated activation of GSK3β in amyotrophic lateral sclerosis (ALS). ALS-associated genes, such as SOD1, contribute to the increased activation of GSK3β, which is implicated in forming cytoplasmic aggregates of toxic proteins.

Furthermore, the interplay between GSK3β and other pathways, such as mTOR, complicates autophagy regulation in ALS. GSK3β can act both upstream and downstream of mTOR, influencing autophagy based on cellular conditions (Lu et al., 2021). mTOR inhibition has been shown to induce autophagy, counteracting neurodegeneration in ALS models (Dey et al., 2017). This dual role emphasizes GSK3β′s therapeutic potential, as modulating its activity could restore autophagy and improve neuronal health in ALS (Kong et al., 2019). Therefore, GSK3β is a crucial autophagy regulator in ALS, affecting toxic protein aggregate clearance and neuronal survival. Modulating GSK3β activity offers a promising strategy to enhance autophagy and potentially lessen the neurodegenerative effects of ALS (Figure 8).

## 9 Therapeutic approaches targeting GSK3β to modulate autophagy in NDs

For their therapeutic potential in diseases such as neurodegenerative disorders and cancer, GSK3β inhibitors have undergone extensive preclinical and clinical evaluation. Because GSK3β regulates key cellular processes including autophagy, cell survival, and apoptosis, these inhibitors hold promise for modulating disease pathways. In preclinical studies, several small-molecule GSK3β inhibitors have been identified and characterized. Compounds such as CHIR-99021, CHIR-98014 and SB216763 have been utilized in various cellular and animal models to explore their effects on GSK3β activity and associated disease mechanisms (Hua et al., 2023). These studies have demonstrated that GSK3β inhibition can lead to reduced tau protein phosphorylation in models of Alzheimer’s disease, suggesting a potential therapeutic benefit in neurodegenerative contexts (Zhao et al., 2024). Additionally, the use of lithium, a well-known GSK3β inhibitor, has shown promise in enhancing neuronal survival and promoting neurogenesis in models of neurodevelopmental disorders (Fuchs et al., 2014). Furthermore, the development of ATP non-competitive inhibitors of GSK3β has been explored as a strategy to improve selectivity and efficacy. These inhibitors bind to unique domains within GSK3β, potentially normalizing its function rather than completely inhibiting it (Liang Z. et al., 2016). This approach may mitigate the adverse effects associated with complete GSK3β inhibition, which can disrupt essential signaling pathways (Table 1).

**TABLE 1:** Classification of GSK3β inhibitors based on mechanism of action, including ATP-competitive and non-ATP-competitive inhibitors, and their corresponding chemical structures.

Type	Class	Compound	IUPAC name	Chemical structure	References
ATP competitive	Indoles	BIO, indirubin-3′-oxime	3-(hydroxyamino)-1H,2′H-[2,3′-biindol]-2′-one	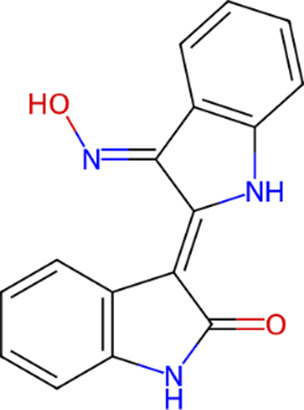	Leclerc et al. (2001)
	Maleimides	SB-216763	3-(2,4-Dichlorophenyl)-4-(1-methyl-1H-indol-3-yl)-1H-pyrrole-2,5-dione	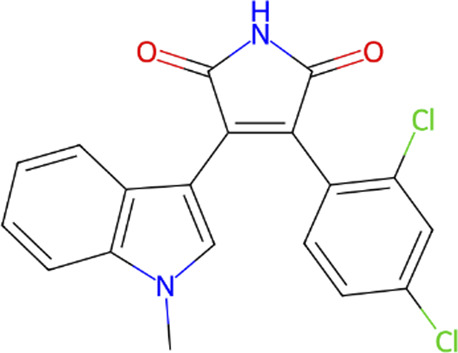	Coghlan et al. (2000)
		SB-415286	3-[(3-Chloro-4-hydroxyphenyl) amino]-4-(2-nitrophenyl)-1H-pyrrole-2,5-dione	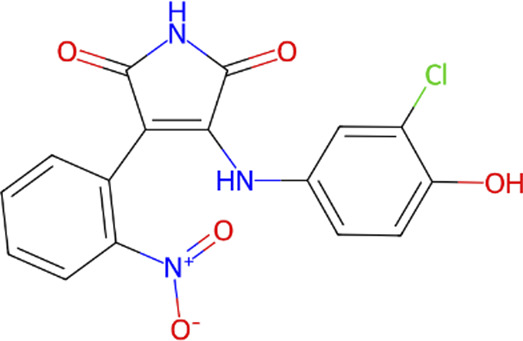	Coghlan et al. (2000)

		BIP-135	3-(5-bromo-1-methyl-1H-indol-3-yl)-4-(benzofuran-3-yl) pyrrole-2,5-dione	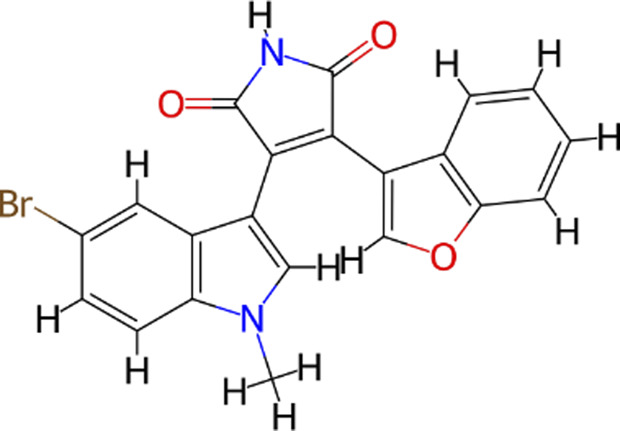	Pandey and DeGrado (2016), Saraswati et al. (2018)
	Pyridines and pyrimidines	CHIR98014	6-N-[2-[[4-(2,4-dichlorophenyl)-5-imidazol-1-ylpyrimidin-2-yl] amino] ethyl]-3-nitropyridine-2,6-diamine	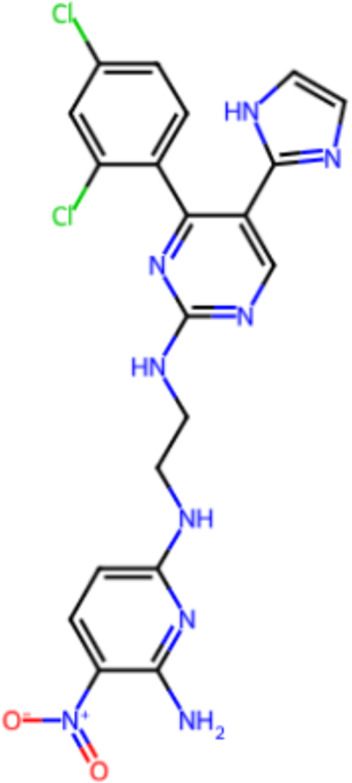	Ring et al. (2003)

		CHIR98023	*N*'-[4-(2,4-dichlorophenyl)-5-(1*H*-imidazol-2-yl) pyrimidin-2-yl]-*N*-(5-nitropyridin-2-yl) ethane-1,2-diamine	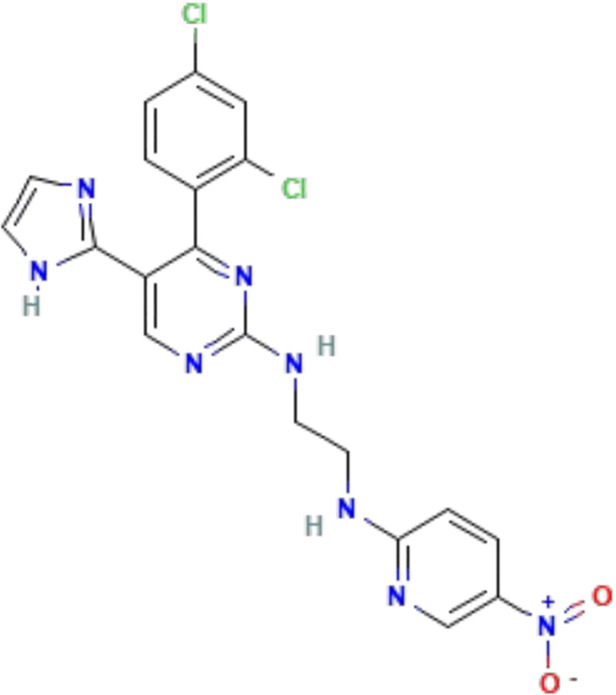	Ring et al. (2003)
		CHIR99021	6-((2-((4-(2,4-Dichlorophenyl)-5-(4-methyl-1H-imidazol-2-yl) pyrimidin-2-yl) amino) ethyl) amino) nicotinonitrile	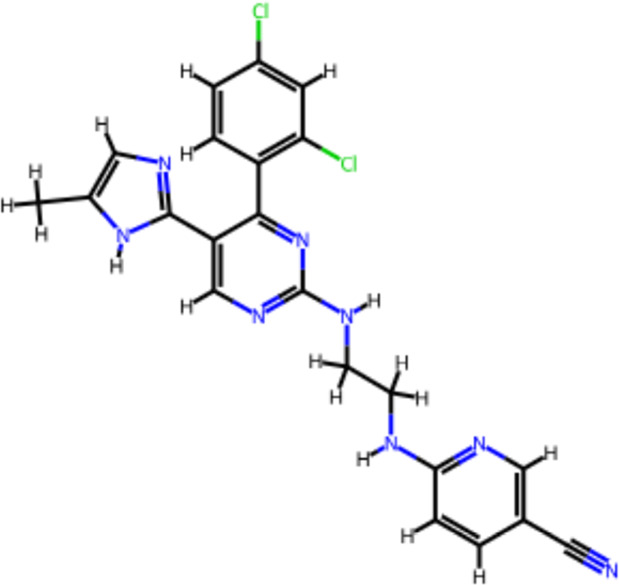	Ring et al. (2003)

		AZD1080	(3E)-3-[5-(morpholin-4-ylmethyl)-1H-pyridin-2-ylidene]-2-oxo-1H-indole-5-carbonitrile	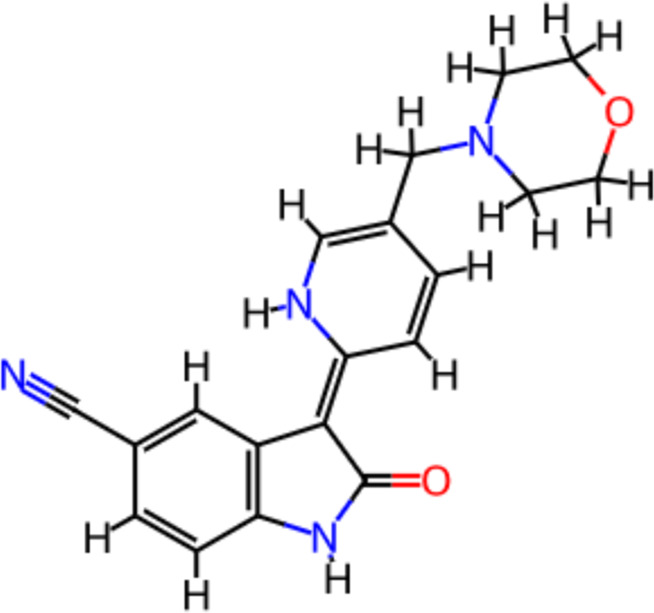	Peat et al. (2004), Bhat et al. (2018), Saraswati et al. (2018), Griebel et al. (2019)
		SAR502250	2-[(2*S*)-2-(4-fluorophenyl) morpholin-4-yl]-3-methyl-6-pyrimidin-4-ylpyrimidin-4-one	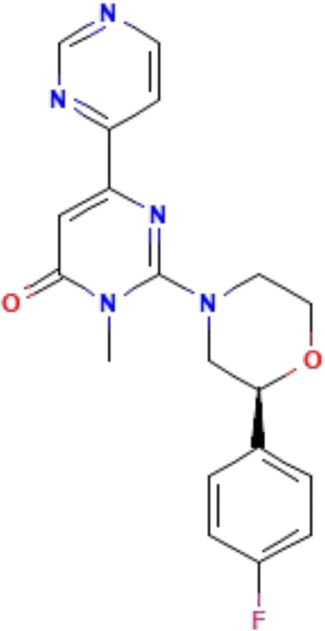	Ring et al. (2003)
		IMID1	Pomalidomide, is 4-amino-2-(2,6-dioxopiperidin-3-yl) isoindole-1,3-dione	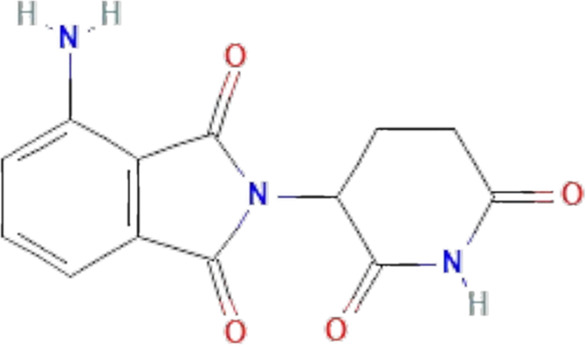	Ring et al. (2003)

		TWS119	3-[[6-(3-aminophenyl)-7H-pyrrolo[2,3-d] pyrimidin-4-yl] oxy] phenol	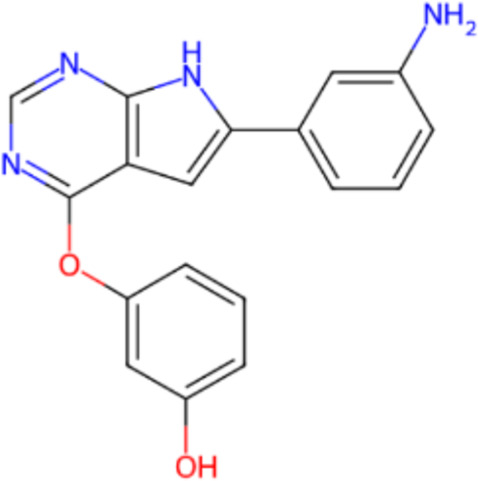	Ring et al. (2003)
		JGK-263	6-chloro-8-(3-pyridin-4-ylpropylamino)-2*H*-[1,2,4] triazolo[4,3-a]pyridin-3-one	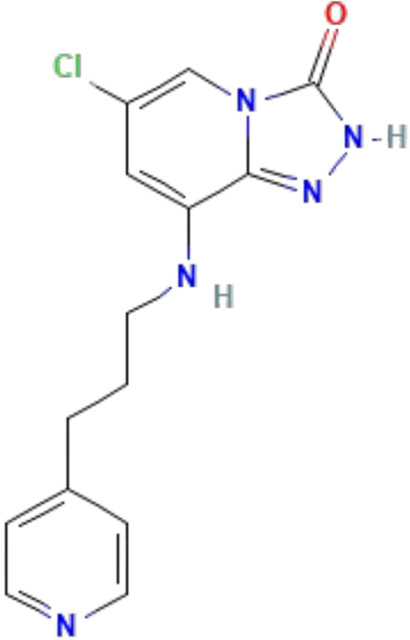	Ring et al. (2003)

	Thiazole	AR-A014418	1-[(4-methoxyphenyl) methyl]-3-(5-nitro-1,3-thiazol-2-yl) urea	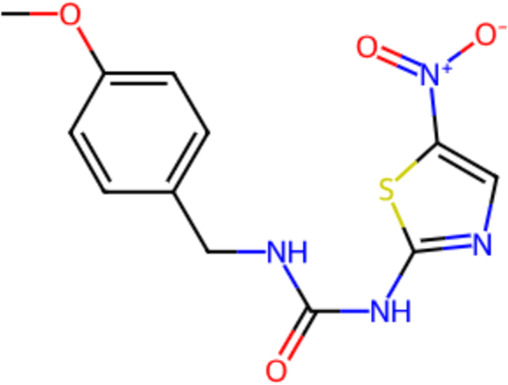	Bhat et al. (2018)

	Paullones	Kenpaullone	9-bromo-7,12-dihydro-5H-indolo[3,2-d] benzazepin-6-one	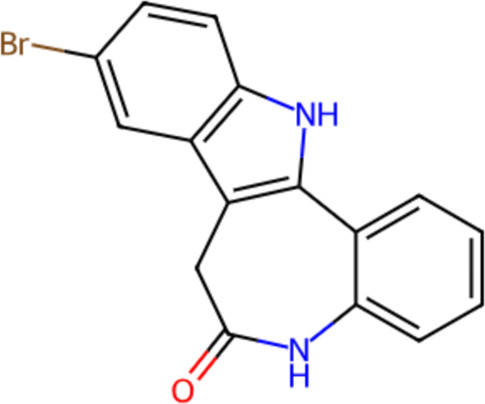	Kunick et al. (2004), Stukenbrock et al. (2008)
		Alsterpaullone	9-nitro-7,12-dihydro-5H-indolo[3,2-d]benzazepin-6-one	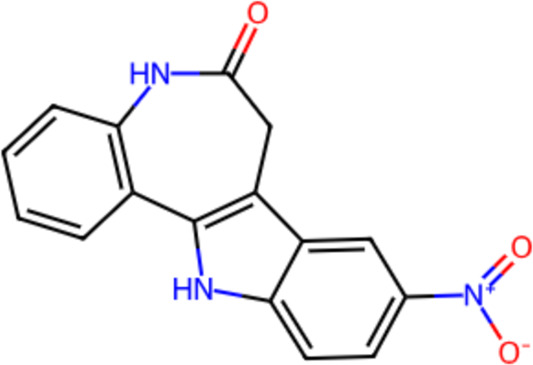	Kunick et al. (2004), Stukenbrock et al. (2008); Kunick et al. (2004); Stukenbrock et al., 2008)
		Cazpaullone	6-Oxo-5,6,7,12-tetrahydropyrido[3′,2′:2,3]azepino[4,5-b]indol-9-carbonitril	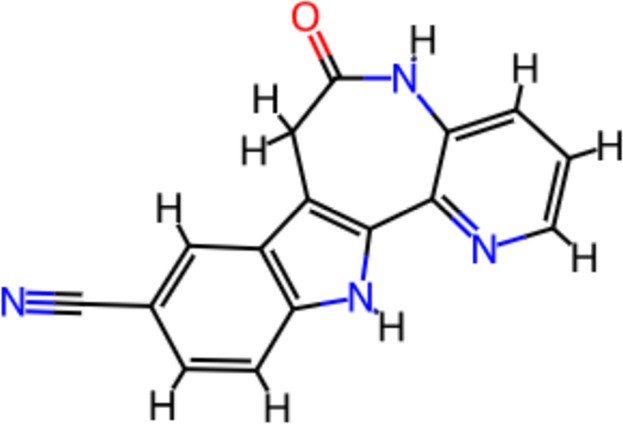	

		Azakenpaullone	14-bromo-3,8,18-triazatetracyclo[9.7.0.02,7.012,17]octadeca-1(11),2(7),3,5,12(17),13,15-heptaen-9-one	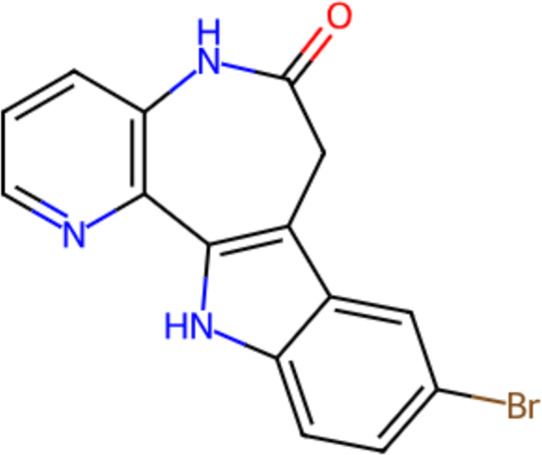	Kunick et al. (2004), Stukenbrock et al. (2008)
	Pyrazine	AZD2858	3-amino-6-[4-(4-methylpiperazin-1-yl) sulfonylphenyl]-N-pyridin-3-ylpyrazine-2-carboxamide	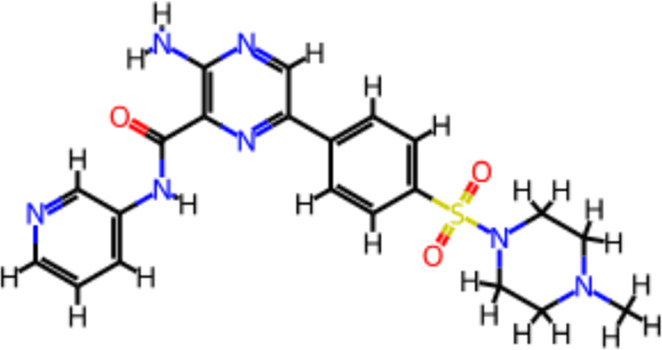	Bhat et al. (2018)
	Oxadiazole	MMBO	2-(Methylamino)isobutyric acid	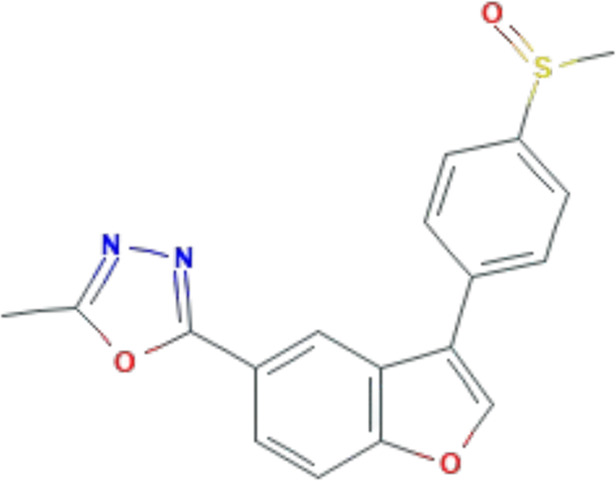	Onishi et al. (2011)

		TCS2002	2-Methyl-5-[3-[4-(methylsulfinyl)phenyl]-5-benzofuranyl]-1,3,4-oxadiazole	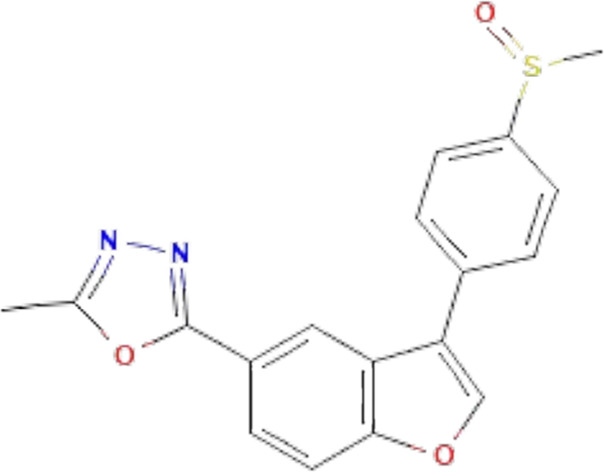	Onishi et al. (2011)
	Oxazole-carbaxamide	PF-04802367 (PF-367)	N-(3-(1H-1,2,4-triazol-1-yl)propyl)-5-(3-chloro-4-methoxyphenyl)oxazole-4-carboxamide	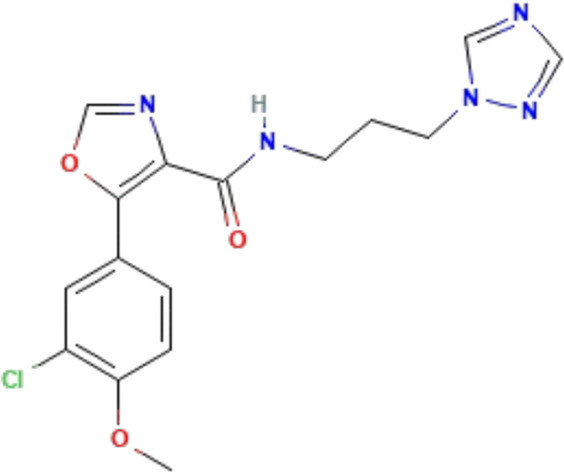	Liang et al. (2016a)
	Pyrazolodihydropyridine	BRD0705	(4S)-4-ethyl-7,7-dimethyl-4-phenyl-2,6,8,9-tetrahydropyrazolo[3,4-b]quinolin-5-one	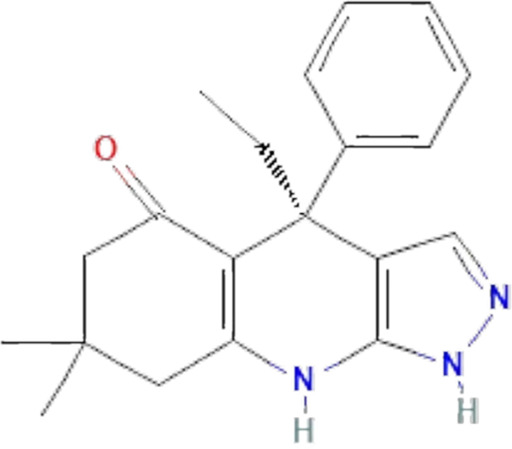	Wagner et al. (2016)

		BRD3731	(6-(4-Chlorophenyl)-3-(4-fluorophenyl)-4-(3-methylphenyl)-1,2,3,4-tetrahydroquinazolin-2-yl)(4-methylphenyl)methanone	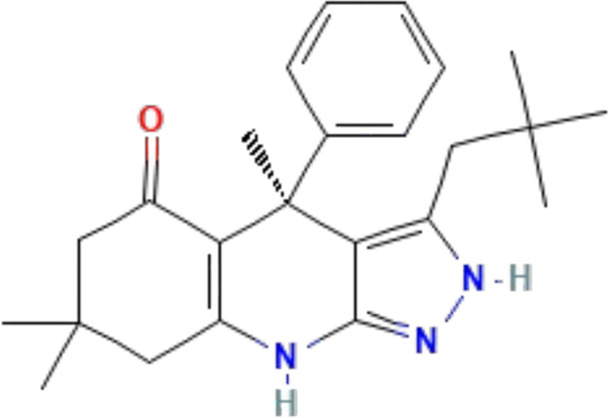	Wagner et al. (2016)
Non-ATP competitive	Thiadiazolidinones	TDZD-8	4-benzyl-2-methyl-1,2,4-thiadiazolidine-3,5-dione	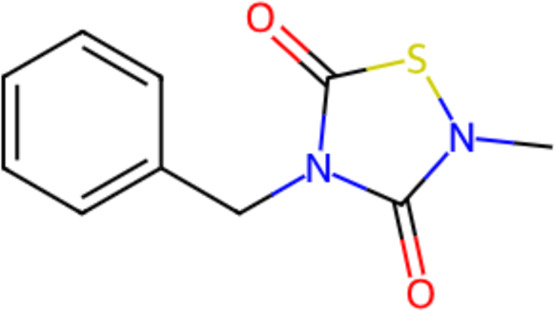	Martinez et al. (2002), Palomo and Martinez (2017)
		Tideglusib (NP031112)	2-(1-naphthalenyl)-4-(phenylmethyl)-1,2,4-thiadiazolidine-3,5-dione	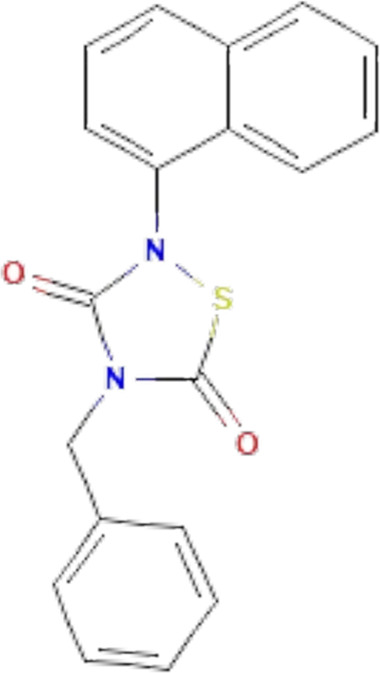	Martinez et al. (2002), Palomo and Martinez (2017)

	Chloromethyl thienyl and halomethyl phenyl ketones	GSK-3β Inhibitor VI	2-Chloro-1-(4,5-dibromo-thiophen-2-yl)-ethanone	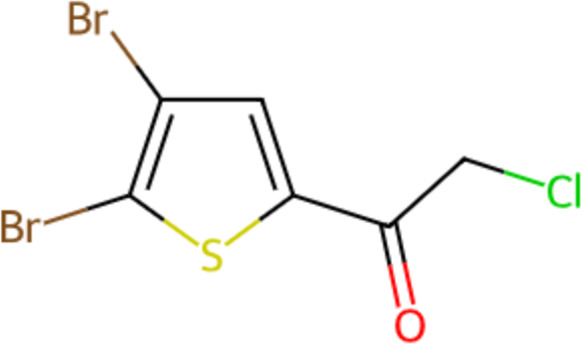	Conde et al. (2003)
Substrate competitive	Iminothiadiazoles	5-imino-1,2,4-thiadiazoles	—	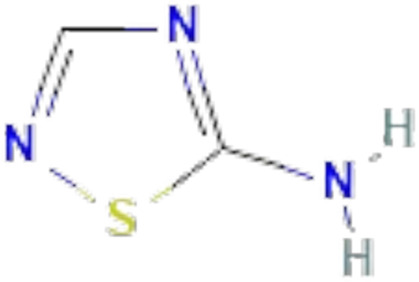	Palomo et al. (2012)

Clinically, GSK3β inhibitors have been assessed for safety and efficacy in various conditions. The selective GSK3β inhibitor TDZD-8 has been used in trials to evaluate its effects on cognition and neuroprotection in neurodegenerative disease patients (Dey et al., 2017). The results indicated that TDZD-8 could effectively modulate GSK3β activity, leading to improvements in cognitive outcomes. Moreover, tideglusib, another GSK3β inhibitor, has been investigated for its potential in treating conditions such as Alzheimer’s disease and cancer. Tideglusib has demonstrated therapeutic potential in preclinical studies by reducing toxic protein aggregates and promoting autophagy (Swinney et al., 2016). Clinical trials have also demonstrated its safety profile, making it a candidate for further investigation in neurodegenerative disorders (Yan et al., 2019). At the molecular level, these therapeutic effects are linked to GSK3β′s regulation of autophagic proteins. For example, activation of GSK3β has been associated with reduced conversion of LC3B-I to LC3B-II, thereby impairing autophagosome formation, while its inhibition increases LC3B-II levels and enhances clearance of aggregated proteins (Sun et al., 2016; Vergoten et al., 2022; Abu-Elfotuh et al., 2023). Similarly, Beclin-1, which plays a key role in autophagy initiation through complex formation with PI3K, is positively regulated upon GSK3β inhibition, restoring autophagic activity and reducing neuronal loss in neurodegenerative disease models (Joshi et al., 2017; Li et al., 2019; Abu-Elfotuh et al., 2023). Consequently, GSK3β inhibitors represent a promising therapeutic strategy in both preclinical and clinical settings. Their ability to modulate autophagy and influence key signaling pathways makes them valuable candidates for treating neurodegenerative diseases and other conditions. Ongoing research continues to refine these inhibitors and explore their full therapeutic potential.

Combining therapies that target GSK3β and autophagy pathways is a promising strategy for treating neurodegenerative diseases and other conditions. By simultaneously modulating these pathways, researchers aim to enhance therapeutic efficacy and improve patient outcomes. Because GSK3β is a crucial autophagy regulator, inhibiting it increases autophagic activity, essential for removing toxic protein aggregates associated with neurodegenerative diseases such as Alzheimer’s disease, Parkinson’s disease and Huntington’s disease (Sun et al., 2016; Weikel et al., 2016). One combination therapy strategy uses GSK3β inhibitors with agents that activate autophagy or enhance related signaling. AMPK activation further enhances autophagy when GSK3β is inhibited. This is supported by evidence that AMPK activation inactivates GSK3β, lifting its inhibitory effect on LC3B and Beclin-1, thereby amplifying autophagic clearance and protecting neurons under conditions of proteotoxic stress (Yang et al., 2010; Duan et al., 2019). This combination may be particularly beneficial in metabolic diseases, where GSK3β inhibition increases autophagy while AMPK activation reduces inflammation and oxidative stress (Weikel et al., 2016). Such synergistic effects could improve the prognosis for patients with diabetic cardiovascular complications and other metabolic disorders. Preclinical studies have explored combining curcumin with GSK3β inhibitors. Curcumin enhances the neuroprotective effects of hUC-MSC transplantation by modulating the Akt/GSK3β pathway, improving anti-inflammatory responses and neuronal recovery after ischemic injury (Li et al., 2023). This highlights the potential of combining natural compounds with GSK3β inhibitors to enhance therapeutic outcomes.

Furthermore, the interplay between GSK3β and the Wnt/β-catenin signaling pathway constitutes an additional therapeutic opportunity for combination therapies. The inhibition of GSK3β can lead to β-catenin stabilization, which in turn promotes autophagy and may potentially mitigate tumor progression in cancerous conditions (Qin et al., 2022). For example, emodin has been shown to downregulate GSK3β phosphorylation, thereby enhancing autophagic degradation of oncogenic proteins (Qin et al., 2022). This suggests that targeting both GSK3β and autophagy could provide a dual benefit in cancer treatment. In the context of cardiovascular health, compounds like spinosin have demonstrated the ability to inhibit GSK3β phosphorylation, leading to increased basal autophagy via the AMPK pathway. This combination has shown protective effects against myocardial ischemia and reperfusion injury (Gu et al., 2019). Such findings underscore the importance of exploring combination therapies that target both GSK3β and autophagy pathways to enhance cardio protection. Furthermore, the dual inhibition of GSK3β and the PI3K/mTOR pathway has been investigated as a potential therapeutic strategy in cancer. By combining autophagy inhibitors with PI3K/mTOR inhibitors, researchers have observed enhanced tumor cell apoptosis, indicating a synergistic effect that could overcome resistance mechanisms in cancer cells (Huang et al., 2022). This approach could be adapted for neurodegenerative diseases, where similar resistance mechanisms may exist. Similarly, targeting GSK3β to restore Beclin-1 expression and LC3B-II conversion in neuronal models may offer a translatable strategy for neurodegenerative diseases, where impaired autophagic flux is a major pathological driver (Joshi et al., 2017; Sophia et al., 2018). Thus, combination therapies targeting GSK3β and autophagy pathways hold significant promise for enhancing therapeutic efficacy in various diseases. By utilizing the synergistic effects of these pathways, researchers can develop more effective treatment strategies for neurodegenerative diseases, metabolic disorders, and cancer.

## 10 Challenges in targeting GSK3β for autophagy modulation

Targeting GSK3β for autophagy modulation presents several challenges that can complicate therapeutic strategies in neurodegenerative diseases and other conditions. While GSK3β inhibitors have shown promise in enhancing autophagic processes, their application is often hindered by the complexity of role of GSK3β in cellular signaling and the potential for off-target effects. One significant challenge is the dual role of GSK3β in regulating autophagy. The influence of GSK3β on autophagic processes is context-dependent, with the enzyme capable of both promoting and inhibiting autophagy and the specific signaling pathways involved. For example, while inhibition of GSK3β has been shown to enhance autophagy in certain models, it can also lead to dysregulation of other pathways, including mTOR pathway that is critical for autophagy regulation (Ren et al., 2016). This complexity necessitates a careful balance in modulating GSK3β activity to avoid adverse effects on cellular homeostasis. Moreover, the phosphorylation status of GSK3β plays a crucial role in its activity. Phosphorylation at tyrosine 216 (Tyr216) results in the activation of GSK3β, whereas phosphorylation at serine 9 (Ser9) leads to its inhibition (Yang et al., 2012; Krishnankutty et al., 2017). This intricate regulation means that simply inhibiting GSK3β may not yield the desired outcomes if the phosphorylation dynamics are not properly understood and managed. For instance, certain inhibitors may lead to an increase in non-phosphorylated GSK3β, which could have unintended consequences on cellular functions (Krishnankutty et al., 2017).

Another challenge lies in the specificity of GSK3β inhibitors. Many compounds that inhibit GSK3β can also affect other kinases, leading to off-target effects that complicate the interpretation of results equally in preclinical and clinical studies (Phukan et al., 2010; Pal et al., 2014). This lack of specificity can result in unwanted side effects, particularly in the central nervous system (CNS), where GSK3β is associated with numerous physiological processes. The development of more selective GSK3β inhibitors is essential to minimize these off-target effects and enhance therapeutic efficacy (Pandya et al., 2024). Additionally, the potential for compensatory mechanisms in cellular signaling pathways poses another challenge. Inhibition of GSK3β may activate alternative pathways that could counteract the intended effects of autophagy modulation. The activation of β-catenin signaling, which occurs upon GSK3β inhibition, can lead to the transcription of genes that may promote cell survival but also contribute to tumorigenesis in certain contexts (McCubrey et al., 2014). This dualistic nature underscores the necessity of a comprehensive understanding of the implicated signaling networks in order to accurately predict the consequences of GSK3β modulation. Lastly, the timing and duration of GSK3β inhibition are critical factors that can influence the effectiveness of autophagy modulation. Chronic inhibition of GSK3β may lead to long-term alterations in cellular signaling that could be detrimental, particularly in the context of neurodegenerative diseases where precise regulation of cellular processes is vital (Choi et al., 2020). Therefore, the development of strategies that allow for temporal control of GSK3β activity could enhance the therapeutic potential of combination therapies targeting autophagy. Hence, while targeting GSK3β for autophagy modulation holds significant promise for treating neurodegenerative diseases and other conditions, several challenges must be addressed. These include the dual role of GSK3β in autophagy regulation, the complexity of its phosphorylation dynamics, the specificity of inhibitors, compensatory signaling mechanisms and the timing of intervention. A deeper understanding of these factors will be essential for the successful development of GSK3β-targeted therapies.

## 11 Future directions for drug development

Future directions for drug development targeting GSK3β and its role in various diseases, particularly neurodegenerative disorders and cancer are promising yet complex. The multifaceted nature of GSK3β in involvement in cellular signaling pathways necessitates a nuanced approach to drug design and therapeutic strategies. One significant area of focus is the development of selective GSK3β inhibitors. Current inhibitors, such as CHIR-99021 and SB216763, have shown efficacy in preclinical models but are often classified as toolkit compounds owing to their non-specificity and potential for off-target effects (Hua et al., 2023). Future investigations should be directed toward the identification and refinement of novel compounds with high selectivity for GSK3β inhibition, minimizing off-target effects on other kinases. Sophisticated computational approaches, including virtual screening and molecular dynamics simulations, can facilitate the design and development of these selective inhibitors (Zhu et al., 2020). By understanding the molecular interactions and conformational dynamics of GSK3β, researchers can develop drugs that specifically target this kinase while minimizing side effects.

Additionally, combination therapies that target GSK3β alongside other pathways could enhance therapeutic efficacy. For instance, combining GSK3β inhibitors with agents that activate autophagy or inhibit the mTOR pathway may provide synergistic effects, particularly in neurodegenerative diseases where autophagic dysfunction is prevalent (Rodríguez-Urgellés et al., 2022). These strategies could enhance the clearance of toxic protein aggregates like amyloid-beta in Alzheimer’s disease or mutant huntingtin in Huntington’s disease, thus reducing neurodegeneration. GSK3β′s role in cancer progression also offers drug development opportunities, particularly in aggressive cancers like triple-negative breast cancer, where it is linked to EMT and cancer stem cell properties (Vijay et al., 2019). Targeting GSK3β in combination with existing cancer therapies may enhance treatment responses and overcome resistance mechanisms. For example, studies have shown that GSK3β inhibition can sensitize cancer cells to chemotherapeutic agents, suggesting that this approach could be beneficial in clinical settings (Ugolkov et al., 2018).

Moreover, the exploration of role of GSK3β in viral infections, such as COVID-19, opens new avenues for drug development. Inhibitors of GSK3β have demonstrated potential in impairing viral replication by affecting the phosphorylation of viral proteins (Liu et al., 2021; Shapira et al., 2022). This recommends that GSK3β inhibitors could be repurposed or developed as antiviral agents, particularly in the context of emerging infectious diseases. Furthermore, understanding the cellular context in which GSK3β operates is crucial for effective drug development. Research has shown that effects of GSK3β can vary significantly depending on the cell type and the specific signaling pathways activated (Scala et al., 2018). Therefore, future studies should focus on elucidating the cell-type-specific roles of GSK3β to design therapies that maximize therapeutic benefits while minimizing adverse effects. Hence, future directions for drug development targeting GSK3β should emphasize the design of selective inhibitors, the investigation of combination therapies and the investigation of role of GSK3β in various diseases, including cancer and viral infections. By utilizing advanced computational techniques and understanding the complex signaling networks involving GSK3β, researchers can develop innovative therapeutic strategies that hold promise for treating a range of conditions.

## 12 Emerging areas of research and knowledge gaps

Emerging areas of research surrounding GSK3β highlight significant knowledge gaps that warrant further investigation. As GSK3β is implicated in various physiological and pathological processes, understanding its multifaceted roles can lead to novel therapeutic strategies for neurodegenerative diseases, cancer and other conditions. One promising area of research is the exploration of role of GSK3β in neuroinflammation and neuroprotection. Studies suggest that inhibiting GSK3β enhances the neuroprotective effects of hUC-MSC transplantation, particularly through the AKT/GSK3β/β-TrCP/Nrf2 signaling axis (Li et al., 2023). However, the mechanisms by which GSK3β modulates neuroinflammation are not well understood. Future research should focus on elucidating the signaling pathways involved in GSK3β-mediated neuroprotection and how these pathways can be effectively targeted in therapeutic contexts.

GSK3β has a dual role in cancer, acting as both a tumor suppressor and promoter. For example, in oral squamous cell carcinoma, Ser9 phosphorylation inactivates GSK3β, promoting oncogenic signaling (Velmurugan et al., 2020). This duality complicates the development of GSK3β inhibitors as cancer therapies. Further studies are needed to clarify the contexts in which GSK3β acts as a tumor promoter *versus* a suppressor, which could inform the design of more effective cancer treatments (Augello et al., 2020). Moreover, the integration of GSK3β inhibitors in cancer immunotherapy presents a novel frontier. While immune checkpoint inhibitors (ICIs) have shown promise, their effectiveness is often limited by resistance mechanisms (Augello et al., 2020). Investigating the potential of GSK3β inhibitors to enhance the efficacy of ICIs could lead to improved outcomes for patients with various cancers. Understanding how GSK3β interacts with immune signaling pathways will be crucial in this regard.

Additionally, the role of GSK3β in metabolic disorders, particularly its interaction with the PI3K/AKT signaling pathway, remains an area for exploration. GSK3β activity is negatively regulated by AKT and its dysregulation has been associated with conditions like diabetic encephalopathy (Chu et al., 2025). Future studies should aim to clarify the mechanisms by which GSK3β influences metabolic processes and how these interactions can be designed for therapeutic benefit. Furthermore, the relationship between GSK3β and oxidative stress is another emerging area of interest. Oxidative stress has been shown to activate GSK3β, contributing to neuronal damage (Ganesan et al., 2021). Investigating the interaction between GSK3β, oxidative stress and neurodegenerative processes could yield valuable insights into potential therapeutic targets for diseases characterized by oxidative damage. Lastly, the structural biology of GSK3β and the development of novel inhibitors remain critical research areas. Understanding the conformational dynamics of GSK3β, particularly in its inactive (DFG-out) state, can inform the design of more selective inhibitors that minimize off-target effects (Balasubramaniam et al., 2020). Although significant progress has been made in understanding GSK3β′s role in various diseases, further research is needed on the pharmacological properties of GSK3β inhibitors, particularly their specificity and mechanism of action, to advance therapeutic applications. Future research should focus on elucidating the complex signaling networks involving GSK3β, exploring its dual roles in cancer, integrating GSK3β inhibitors into immunotherapy and developing novel, selective inhibitors. The therapeutic potential of GSK3β modulation in clinical settings cannot be fully realized without addressing these knowledge gaps.

## 13 Conclusion

This review focuses on the pivotal role of GSK3β in regulating autophagy and the resulting implications for neurodegenerative diseases, including Alzheimer’s disease, Parkinson’s disease, Huntington’s disease and ALS. By influencing key pathways and autophagy-related proteins, GSK3β serves as a critical mediator of cellular homeostasis and neurodegeneration. Targeting GSK3β through selective inhibitors and combination therapies offers a promising approach to restore autophagic activity, reduce toxic protein aggregates and protect neuronal health. However, the complex involvement of GSK3β in multiple signaling pathways presents challenges requiring careful therapeutic modulation. Future research should focus on developing blood-brain barrier (BBB) permeable, selective GSK3β inhibitors and investigating emerging targets such as mitochondrial autophagy and non-coding RNAs. The advancement of these strategies will provide a pathway for the development of novel therapeutic interventions to combat neurodegenerative pathologies and enhance patient outcomes.
